# A weak health response is increasing the risk of excess mortality as food crisis worsens in Somalia

**DOI:** 10.1186/s13031-017-0114-0

**Published:** 2017-07-03

**Authors:** Andrew Seal, Francesco Checchi, Nancy Balfour, Abdi-Rashid Haji Nur, Mohamed Jelle

**Affiliations:** 1 Institute for Global Health, UCL, London, UK; 2 Epidemiology and International Health, London School of Hygiene and Tropical Medicine, London, UK; 3 Centre for Humanitarian Change, Nairobi, Kenya; 4 Concern Worldwide, Mogadishu, Somalia

Since 1991, Somalia has been afflicted by conflict involving factions within the country, regional powers, and international actors. Following a year of failed rains the current humanitarian situation in Somalia is extremely serious. Early warning alerts have been issued and the UN has launched an appeal aiming to prevent the onset of famine and its defining features of extreme food insecurity, malnutrition, and excess mortality [1]. Large scale distress migration has been reported with 599,000 people moving due to the crisis since November 2016, including 59,000 in the first 2 weeks of April [2].

While the focus of the response is understandably on food assistance (including cash transfers) the health situation is equally alarming. Infectious diseases are major causes of excess mortality under the famine-like conditions that are expected over the next few months [3].

Chronic insecurity has contributed to a degraded health system and humanitarian actors have become important providers of health services. Following the withdrawal of MSF in 2013 and problems in implementing alternative programmes, the health system within Somalia, particularly southern Somalia, has severe capacity constraints [4].

Water availability in southern Somalia is critical following the drying of the Shabelle River in January. While the Gu rainy season has now started, extensive damage to crops, pasture and livelihoods has already occurred and the adequacy and extent of rainfall remains uncertain [5]. An outbreak of cholera is spreading rapidly with 28,408 cases and 558 deaths reported this year up to April 16th and this is likely to accelerate with the onset of the Gu rains [6]. An oral cholera vaccination campaign was announced by WHO and the MOH on March 15th. However, the speed at which this can be implemented may not contain the outbreak. Problems of capacity, funding, and access are limiting the scale up of standard and effective responses, including water, sanitation, and hygiene activities to control the spread of cholera.

Measles vaccination coverage within Somalia is generally poor. A large outbreak of measles took place in Kismayo at the end of 2016 and newly available verbal autopsy data indicate that measles has caused 9% of deaths in displaced children in Mogadishu during this pre-famine stage [7]. Measles is an important cause of death in famine and any residual herd immunity from the widespread outbreaks in 2011 will have waned by now due to the build-up of a susceptible pool of unvaccinated children under 5 y – i.e. the likely famine event may unfortunately coincide with the normal cycle of measles outbreaks in such settings. While Somalia lacks a centralised measles surveillance system, the latest media report indicate 5700 suspected cases since the start of the year [8]. A vaccination campaign was belatedly announced in Baidoa on April 25th but is designed to have limited geographical coverage, excluding many areas where internally displaced people are known to be concentrating [9].

As of April 3rd, the Somalia Health Cluster reported that only 7% % of the $US 85 million requested for health interventions had been provided [10]. Somalia is an extremely difficult operating environment but WHO needs to rapidly enhance its humanitarian health coordination role and partners need to effectively scale up public health interventions, particularly vaccination.

**Fig. 1:**
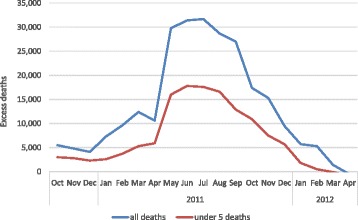
Estimated excess mortality by month during the 2011 famine in Somalia

Famine occurred in southern Somalia in 2011 and caused the deaths of an estimated 258,000 people (Fig. 1) [11]. 2017 could be worse and a more effective health response is urgently needed.
